# Efficacy and safety of catheter ablation for atrial fibrillation in patients with history of cancer

**DOI:** 10.1186/s40959-023-00171-4

**Published:** 2023-04-05

**Authors:** Sarju Ganatra, Sonu Abraham, Ashish Kumar, Rohan Parikh, Rushin Patel, Sumanth Khadke, Amudha Kumar, Victor Liu, Andrea Nathalie Rosas Diaz, Tomas G. Neilan, David Martin, Bruce Hook, Sourbha S. Dani, Aarti Asnani, Anju Nohria

**Affiliations:** 1 Cardio-Oncology Program, Division of Cardiovascular Medicine, Department of Medicine, Lahey Hospital & Medical Center, 41 Mall Road, Burlington, Burlington, MA 01805 USA; 2 Department of Medicine, Cleveland Clinic Akron General, Akron, OH USA; 3 Cardio-Oncology Program, Department of Cardiovascular Medicine, Beth Israel Deaconess Medical Center, Boston, MA USA; 4 Cardiovascular Imaging Research Center (CIRC) and Cardio-Oncology Program, Massachusetts General Hospital, Boston, MA USA; 5 Division of Cardiovascular Medicine, Department of Medicine, Electrophysiology Program, Lahey Hospital & Medical Center, Burlington, MA USA; 6 Department of Cardiovascular Medicine, Electrophysiology Program, Brigham and Women’s Hospital, Boston, MA USA; 7 Cardio-Oncology Program, Department of Cardiovascular Medicine, Brigham and Women’s Hospital, Boston, MA USA

## Abstract

**Background:**

Though the incidence of atrial fibrillation (AF) is increased in patients with cancer, the effectiveness of catheter ablation (CA) for AF in patients with cancer is not well studied.

**Methods:**

We conducted a retrospective cohort study of patients who underwent CA for AF. Patients with a history of cancer within 5-years prior to, or those with an exposure to anthracyclines and/or thoracic radiation at any time prior to the index ablation were compared to patients without a history of cancer who underwent AF ablation. The primary outcome was freedom from AF [with or without anti-arrhythmic drugs (AADs), or need for repeat CA at 12-months post-ablation]. Secondary endpoints included freedom from AF at 12 months post-ablation with AADs and without AADs. Safety endpoints included bleeding, pulmonary vein stenosis, stroke, and cardiac tamponade. Multivariable regression analysis was performed to identify independent risk predictors of the primary outcome.

**Results:**

Among 502 patients included in the study, 251 (50%) had a history of cancer. Freedom from AF at 12 months did not differ between patients with and without cancer (83.3% vs 72.5%, p 0.28). The need for repeat ablation was also similar between groups (20.7% vs 27.5%, p 0.29). Multivariable regression analysis did not identify a history of cancer or cancer-related therapy as independent predictors of recurrent AF after ablation. There was no difference in safety endpoints between groups.

**Conclusion:**

CA is a safe and effective treatment for AF in patients with a history of cancer and those with exposure to potentially cardiotoxic therapy.

## Introduction

Atrial fibrillation (AF) is the most common cardiac arrhythmia affecting 1.5–2% of the general population [1]. The incidence of AF is increased in patients with cancer (up to 20%), particularly among those with advanced age or pre-existing cardiovascular risk factors [2–5]. Postulated mechanisms include atrial remodelling due to a pro-inflammatory state, dysautonomia, paraneoplastic processes, electrolyte abnormalities and direct myocardial damage either due to cancer therapies, surgery or less commonly, metastatic invasion of pulmonary, pericardial and myocardial tissues [6–9]. Certain cancer therapies like Bruton’s tyrosine kinase inhibitors [10], anthracyclines, immune checkpoint inhibitors, antimetabolites, and alkylating agents are associated with a higher incidence of AF [9].

While the presence of AF does not preclude cancer therapy, downstream complications such as thromboembolic events and the development of heart failure (HF) can lead to increased morbidity and mortality in patients with cancer [11]. For example, patients with cancer and AF have a sixfold increased risk of HF and twofold higher risk of stroke compared to the general population [11–13].

In the general population, rhythm control is recommended in patients who are symptomatic despite adequate rate control and in patients with HF [14–17] However, there are several unique considerations for rhythm control in patients with cancer. The co-administration of anti-arrhythmic drugs (AADs) and targeted cancer therapies, especially, tyrosine kinase inhibitors (TKIs), can lead to increased concentrations of either drug, via impaired cytochrome P-450 metabolism or inhibition of P-glycoprotein-mediated transport [18]. An increased propensity for QT prolongation and bradycardia is also present, further limiting the use of AADs in patients with cancer [18].

Catheter ablation for AF is an established treatment modality with relatively high success rates and few procedural complications [19]. Growing evidence demonstrates that catheter ablation for AF in patients with HF is associated with improved clinical outcomes and quality of life compared to medical therapy alone [15]. Patients with cancer, particularly those exposed to potentially cardiotoxic antineoplastic therapies, are at a higher risk of developing AF and subsequent cardiomyopathy and HF and catheter ablation may provide a promising treatment option for selected patients. In addition to symptomatic improvement, catheter ablation may alleviate the need for ongoing AAD therapy thus limiting serious drug-drug interactions. However, catheter ablation for AF may be underutilized in patients with cancer due to a concern for lower success rates attributed to the underlying inflammatory state, continued exposure to potentially cardiotoxic and proarrhythmic agents, and a perceived risk of higher complication rates with invasive therapies in this patient population. In this study, we sought to explore the efficacy and safety of catheter ablation for AF in patients with cancer.

## Methods

### Study oversight

This study was approved by the Institutional Review Boards (IRB) of Lahey Hospital & Medical Center, Beth Israel Deaconess Medical Center, and Brigham and Women’s Hospital. The need for written informed consent was waived by the IRB. All authors reviewed the manuscript and attest to the integrity, accuracy, and completeness of the data.

### Study population and design

This was a retrospective cohort study of consecutive patients who underwent catheter ablation for AF between January 1, 2014 and December 31, 2018. The cohort of interest included patients ≥ 18 years of age, with either active cancer, a history of malignancy 5-years prior to the index ablation procedure, or those with exposure to systemic anthracyclines and/or thoracic radiation therapy any time before the index ablation. Patients with non-melanotic skin cancers were excluded. The control group consisted of an equivalent number of consecutive patients without a current or prior history of cancer who underwent catheter ablation for AF at Lahey Hospital & Medical Center. The patients in the control arm were selected from the more contemporary period of the study till we had sufficient numbers matching the cases. Patients with paroxysmal, persistent, and long-standing persistent AF were included. Patients with valvular AF were excluded.

### Data acquisition

Patient demographics and comorbidities (age, gender, body mass index, smoking history, hypertension, hyperlipidemia, diabetes, obstructive sleep apnea, obstructive coronary artery disease, presence of more than mild valvular heart disease, HF and prior stroke or transient ischemic attack (TIA)) were extracted from the electronic medical record. We also collected data regarding medications used for the management of AF prior to ablation, prior cardioversions, left atrial diameter prior to ablation, and type and modality of ablation. Cancer-specific characteristics such as type of malignancy, presence of metastatic disease, active cancer therapy, systemic chemotherapy or surgery in the 5 years prior to ablation, radiation therapy, anthracycline exposure, cancer status at the time of ablation, and cancer recurrence after ablation were also obtained. The CHA_2_DS_2_-VASc score was used to calculate stroke risk. Anticoagulation with either warfarin or direct oral anticoagulants (DOACs) was also recorded.

### Study endpoints

The primary outcome was freedom from AF [with or without anti-arrhythmic drugs (AADs), or need for repeat CA] at 12-months post-ablation in patients with a history of cancer compared to controls. The secondary endpoints included freedom from AF with, and without AADs at 12 months post-ablation. Safety endpoints assessed peri-procedural complications including bleeding requiring investigation or intervention or transfusion (access and non-access site), pulmonary vein stenosis, stroke, and cardiac tamponade within the first 3 months after ablation. The outcomes were documented through detailed chart review in the electronic health record system.

### Statistical analysis

Categorical data are presented as numbers and percentages, and continuous variables as mean ± standard deviation (SD) or median and interquartile range (IQR). Baseline characteristics and outcomes after catheter ablation for AF were compared between patients with cancer and controls. Student’s t test, Wilcoxon rank-sum test, and chi-squared test or Fisher’s exact test were utilized to compare continuous non-skewed, skewed, and categorical variables, respectively. Multivaribale regression analyses were used to determine adjusted odds ratios and predictors of AF recurrence in patients who underwent catheter ablation. All variables with a *p* value < 0.1 in the univariable analyses, with less than 20% of missingness, were included in the multivariable analyses, after assessing for collinearity. Clinically relevant variables deterimed after review of the prior literuature were also included in the multivariable model and the performance of the model was assessed using the Akaike information criterion (AIC). To assess for collinearity, variance inflation factor (VIF) values were calculated for each predictor variable. Values of VIF exceeding 2.5 were viewed as being indicative of high correlation. Our analysis revealed a VIF range from of 1.19- 2.36. The final estimates were not disproportionately skewed by collinearity among predictors. Results were reported as odds ratios (OR) with 95% confidence intervals (CI). Kaplan–Meier survival curves were used to depict outcomes in patients with and without cancer and were compared using log-rank tests. A two-sided *p* value < 0.05 was considered statistically significant. Data analysis was performed using R version 4.0.3.

## Results

### Patient population

**Fig. 1:**
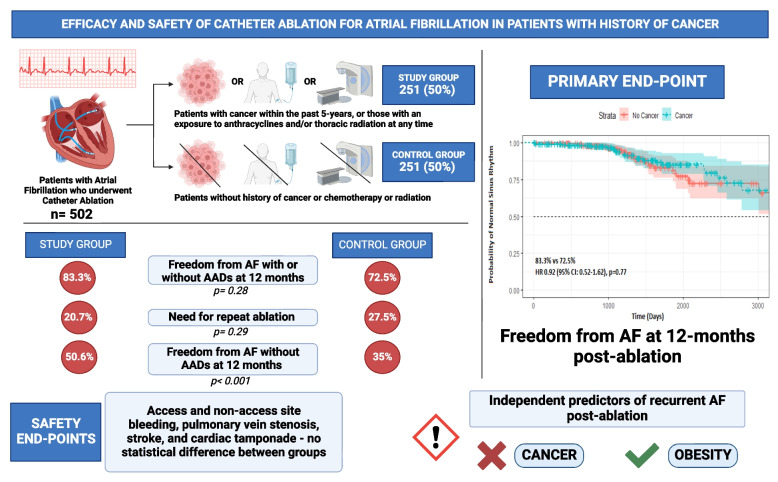
Efficacy and Safety of Catheter Ablation for Atrial Fibrillation in Patients with History of Cancer

The study cohort included 502 patients who underwent catheter ablation for AF. Among these 251 (50%) had a history of cancer (Fig. 1).

### Patient characteristics

Patients with cancer were significantly older than those without cancer (67 vs. 64 years, *p* < 0.01). Sex was balanced between the two groups with 47% females in the cancer group and 42.6% in the non-cancer group (*p* = 0.32). The prevalence of comorbidities including hypertension, diabetes, tobacco use, obstructive sleep apnea, coronary artery disease, valvular heart disease, congestive heart failure, and prior stroke or TIA was similar in both groups. Hyperlipidemia was significantly more common in patients with cancer compared to controls (57% vs 44.6%, *p* = 0.006). There was no difference in overall functional status between the 2 groups (*p* = 0.23) with more than half the patients in each group being categorized as NYHA class 1 (Table 1).

**Table 1:** Baseline Characteristics of patients treated with catheter ablation for atrial fibrillation

	**Cancer (*n* = 251)**	**No Cancer (*n* = 251)**	***P* value**
Age in years, median (IQR)	67 (61–73)	64 (56–70)	< 0.001
Female	118 (47)	107 (42.6)	0.32
Height in meters, median (IQR)	1.73 (1.65–1.80)	1.73 (1.65–1.80)	0.83
Weight in kg, median (IQR)	86.18 (76.10–99.75)	91.4 (76.34–109.35)	0.01
BMI in kg/m^2^, median (IQR)	29 (25–33)	30.19 (26.17–35.25)	0.70
**Comorbidities**			
Hypertension	166 (66.1)	168 (66.9)	0.85
Hyperlipidemia	143 (57)	112 (44.6)	0.006
Diabetes	38 (15.1)	51 (20.3)	0.13
HbA1c %, median (IQR)	5.6 (5.3–6.2)	5.7 (5.3–6.1)	0.86
Obstructive sleep apnea	63 (25.1)	53 (21.1)	0.35
Smoker			0.63
Former	98 (39)	108 (43)	
Current	17 (6.8)	14 (5.6)	
Coronary artery disease	41 (16.3)	36 (14.3)	0.54
Heart failure			0.50
Reduced ejection fraction	44 (17.5)	52 (20.7)	
Preserved ejection fraction	21 (8.4)	16 (6.4)	
NYHA classification			0.23
NYHA 1	136 (54.2)	139 (55.4)	
NYHA 2	78 (31.1)	89 (35.4)	
NYHA 3	25 (10)	14 (5.6)	
NYHA 4	4 (1.6)	1 (0.4)	
Prior Stroke/TIA	20 (8)	14 (5.6)	0.29
**Atrial Fibrillation (AF) Variables**			
Type of AF			0.83
Paroxysmal	136 (54.2)	141 (56.2)	
Persistent	105 (41.8)	103 (41)	
Long standing persistent	9 (3.6)	7 (2.8)	
Prior cardioversion for AF	151 (60.2)	145 (57.8)	0.59
CHA_2_DS_2_-VASc score, median (IQR)	2(1–4)	2(1–3)	0.06
**Drugs used to manage AF before ablation**			
Amiodarone	77 (30.7)	40 (15.9)	< 0.001
Dronedarone	19 (7.6)	12 (4.8)	0.19
Flecainide	74 (29.5)	57 (22.7)	0.08
Dofetilide	43 (17.1)	31 (12.3)	0.13
Propafenone	18 (7.2)	12 (4.8)	0.35
Sotalol	78 (31.2)	68 (27.1)	0.32
Verapamil/Diltiazem	62 (24.7)	54 (21.5)	0.50
Beta Blockers	187 (74.5)	177 (70.5)	0.32
Digoxin	26 (10.4)	22 (8.8)	0.46
Warfarin	105 (41.8)	69 (27.5)	0.002
Direct Oral Anticoagulants	141 (56.2)	146 (58.2)	0.33
**Procedural characteristics**			
Type of ablation			< 0.001
Pulmonary vein isolation	114 (45.4)	154 (61.4)	
Pulmonary vein isolation plus lines	137 (54.6)	97 (38.6)	
Modality of ablation			< 0.001
Radiofrequency ablation	146 (58.2)	21 (8.4)	
Cryoablation	91 (36.2)	134 (53.4)	
Both	14 (5.6)	96 (38.3)	
**Cancer patient specific variables**			
Type of cancer			
Breast carcinoma	75 (29.9)		
Lung carcinoma	15 (6.0)		
Prostate carcinoma	56 (22.3)		
Lymphoma	25 (10.0)		
Other cancer	80 (31.9)		
Metastatic disease	28 (11.2)		
Active treatment at the time of ablation	46 (18.3)		
Systemic chemotherapy in the last 5 years	80 (31.9)		
Surgery for cancer in the last 5 years	114 (45.4)		
Thoracic radiation in the last 5 years			
Left breast	9 (3.6)		
Right breast	40 (15.9)		
Left lung	16 (6.4)		
Right lung	5 (2)		
Anthracycline exposure	41 (16.3)		
Cancer in remission at the time of ablation	205 (81.7)		
Recurrent cancer within a year after ablation	11 (4.4)		
Multiple malignancies	33 (13.1)		

Values are expressed as n (%) unless specified otherwise

*AF* atrial fibrillation, *BMI* body mass index, *NYHA* New York Heart Association, *TIA* transient ischemic attack

The majority of the patients had a diagnosis of paroxysmal AF in both the cancer and controls groups (54.2% vs. 56.2%). Beta blockers were administered equally in those with and without cancer (74.5% vs 70.5%, *p* = 0.32). The most common antiarrhythmic drug utilized in the entire cohort was sotalol (31.2% of cancer patients vs. 27.1% of controls, *p* = 0.32). Amiodarone was used more frequently in cancer patients compared to controls (30.7% vs 15.9%, *p* < 0.001). There was no difference between groups in the utilization of other AADs (Table 1). Cardioversion was attempted prior to ablation in 60.2% of patients with cancer and in 57.8% of those without cancer (*p* = 0.59). The average CHA_2_DS_2_-VASc score in both groups was 2. The majority of patients were on DOACs (56.2% in the cancer group vs. 58.2% in the non-cancer group, *p* = 0.33). In contrast, warfarin use was significantly higher among cancer patients compared to controls (41.8% vs 27.5%, *p* = 0.002) (Table 1).

### Procedural characteristics

There were significant differences between the groups in terms of type and modality of catheter ablation. Radiofrequency ablation was employed more frequently in patients with cancer (58.2% vs. 8.4%, *p* < 0.001). In contrast, cryoablation was used more frequently in controls (53.4% vs 36.2%, *p* < 0.001). The combination of both modalities was also utilized more frequently in controls (38.3% vs 5.6%, *p* < 0.001). Adjuvant substrate modification with linear ablations in addition to pulmonary vein isolation was performed more frequently in patients with cancer compared to controls (54.6% vs 38.6%, *p* < 0.001) (Table 1).

### Cancer characteristics

Among those with cancer, breast cancer was the most common diagnosis (*n* = 50, 19.9%). Other prevalent cancers included prostate cancer (*n* = 33, 13.1%), lymphoma (*n* = 20, 8%) and lung cancer (*n* = 12, 4.8%). A total of 28 (11.2%) patients had metastatic disease and 33 (13.1%) patients had a history of multiple malignancies. Among patients with cancer, 46 (18.3%) were undergoing active treatment and 205 (81.7%) were in remission at the time of catheter ablation. Systemic chemotherapy was administered in 80 (31.9%) patients and surgery for resection of cancer was performed in 114 (45.4%) patients within the 5 years prior to the index ablation. Anthracyclines were utilized in 41 (15.3%) patients. Radiation to the left lung and left breast were performed in 2% and 15.9% of patients, respectively. A total of 11 (4.4%) patients had recurrence of cancer within a year after catheter ablation (Table 1).

## Outcomes

### Primary and secondary outcomes

**Fig. 2:**
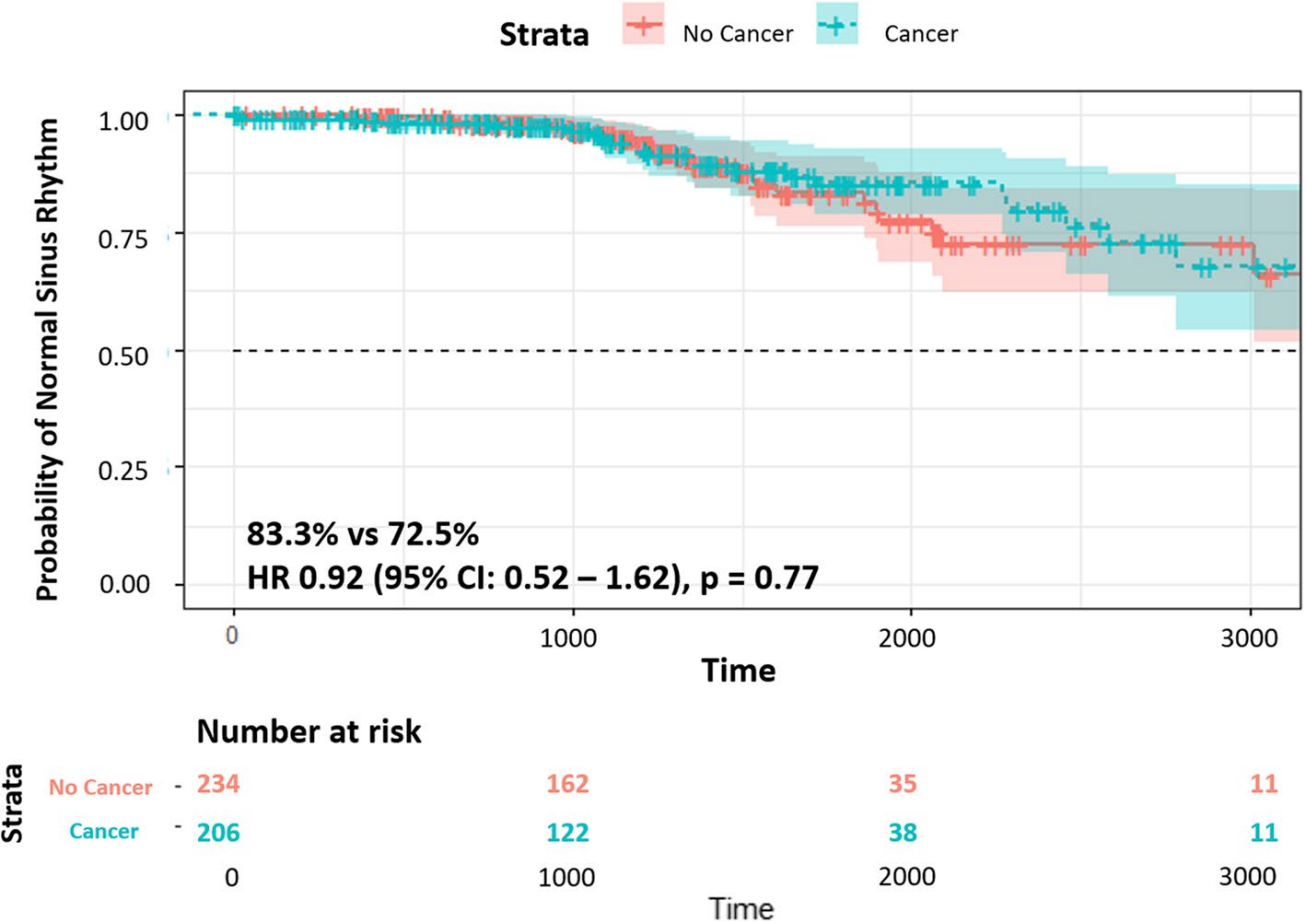
Kaplan–Meier survival curve showing freedom from recurrent atrial fibrillation and need for repeat ablation. There was no significant difference in the freedom from recurrent atrial fibrillation or need for repeat ablation between patients with cancer (green) and non-cancer controls (red) (HR 0.92, 95% CI 0.52–1.62, *p* = 0.77). NSR = normal sinus rhythm

There was no significant difference in the primary outcome of freedom from AF, with or without AADs, or need for repeat ablation at 12 months post-ablation between those with and without cancer (83.3% vs 72.5%, *p* = 0.28) (Table 2) (Fig. 2).

**Table 2:** Outcomes after catheter ablation in patients with and without cancer

	**Cancer (*n* = 251)**	**No Cancer (*n* = 251)**	***P* value**
**Effectiveness Outcomes**			
Recurrence of atrial arrhythmias during the 3-month blanking period	83 (33.1)	78 (31.1)	0.29
Recurrence of atrial arrhythmias during the 3-month blanking period requiring cardioversion	36 (14.3)	38 (15.1)	0.34
Recurrence after 3 months	87 (34.7)	90 (35.8)	0.65
Recurrence after 6 months	77 (30.7)	72 (28.7)	0.43
Recurrence after 12 months	63 (25.1)	57 (22.7)	0.40
Recurrence after 24 months	33 (13.1)	43 (17.1)	0.33
Freedom from AF at 12 months	209 (83.3)	182 (72.5)	0.28
Freedom from AF without AAD at 12 months	127 (50.6)	88 (35.0)	< 0.001
Freedom from AF with AAD at 12 months	82 (32.7)	94 (37.4)	0.19
Need for repeat ablation	52 (20.7)	69 (27.5)	0.29
**Safety Outcomes**			
Bleeding			0.12
Access site	13 (5.2)	6 (2.4)	
Non-access site	7 (2.8)	4 (1.6)	
PV stenosis post ablation	1 (0.4)	2 (0.8)	0.30
Stroke post ablation	4 (1.6)	1 (0.4)	0.15
Cardiac tamponade post ablation	3 (1.2)	2 (0.8)	0.60

All values are expressed as n (%)

*AAD* anti-arrhythmic drugs, *AF* atrial fibrillation, *PV* pulmonary vein

The secondary outcome of freedom from AF without AADs at 12 months was higher in patients with cancer than controls (50.6% vs 35%, *p* < 0.001). In contrast, freedom from AF with AADs at 12 months did not differ between groups (Table 2). The odds of recurrent AF after three, six, nine, and twelve months from the index ablation were similar between groups even after adjusting for multiple clinical covariates and modality of ablation (Table 3). The need for repeat ablation did not differ between the two groups (20.7% of cancer patients vs 27.5% of non-cancer patients, *p* = 0.29). Post-ablation left ventricular ejection fraction was also similar in the cancer and non-cancer groups (60% vs 57%, *p* = 0.31).

**Table 3:** Adjusted odds ratios for recurrence of atrial fibrillation in patients with cancer compared with non-cancer controls

	**OR (95% CI)**	***P* value**	**E value for OR**	**E value for 95% CI**
Recurrence after 3 months	1.02 (0.61–1.70)	0.93	1.11	1
Recurrence after 6 months	1.04 (0.61–1.79)	0.87	1.16	1
Recurrence after 9 months	1.08 (0.60–1.90)	0.80	1.24	1
Recurrence after 12 months	1.05 (0.54–2.06)	0.88	1.18	1

Model adjusted for age, sex, BMI, hypertension, hyperlipidemia, diabetes mellitus, obstructive sleep apnea, coronary artery disease, valvular heart disease, CHA_2_DS_2_-VASc score, type of atrial fibrillation, type of ablation, and modality of ablation

In the entire cohort of patients, obesity (increased BMI) was identified as a significant predictor of recurrent AF after the 3-month post-ablation blanking period with OR 1.07 (95% CI 1.03–1.11, *p* < 0.001) (Table 4). In patients with cancer, increased BMI was also identified as significant predictor of recurrent AF after the 3-month post-ablation blanking period (OR 1.08, 95% CI 1.03–1.15, *p* = 0.002) (Table 5).

**Table 4:** Multivariable regression analysis of the entire study cohort to identify predictors of recurrent atrial fibrillation after a 90-day blanking period

Variables	Odds ratio	*P* value
Age	1.02 (0.99–1.04)	0.24
Female	1.04 (0.66–1.66)	0.86
BMI	1.07 (1.03–1.11)	< 0.001
Cancer	1.02 (0.61–1.70)	0.93
Hypertension	0.72 (0.43–1.21)	0.22
Hyperlipemia	1.31 (0.82–2.10)	0.25
Diabetes	0.86 (0.48–1.53)	0.61
Obstructive sleep apnoea	0.96 (0.57–1.60)	0.87
Coronary artery disease	0.63 (0.35–1.18)	0.15
Aortic stenosis	0.33 (0.17–2.14)	0.32
Paroxysmal atrial fibrillation	0.81 (0.52–1.26)	0.35
Long term persistent atrial fibrillation	1.15 (0.37–3.50)	0.80
CHA_2_DS_2_-VASc score	1.04 (0.84–1.28)	0.71
Pulmonary vein isolation plus lines	0.87 (0.52–1.45)	0.59
Cryoablation	0.74 (0.42–1.30)	0.29
Radiofrequency + Cryoablation	0.86 (0.43–1.73)	0.67

*BMI* body mass index

**Table 5:** Multivariable regression analysis of patients with cancer to identify predictors of recurrent atrial fibrillation after a 90-day blanking period

Variables	OR (95% CI)	*P* value
Age	0.99 (0.94–1.03)	0.62
Female	1.23 (0.52–2.91)	0.64
BMI	1.08 (1.03–1.15)	0.002
Hypertension	0.25 (0.11–0.57)	0.001
Hyperlipemia	1.40 (0.64–3.09)	0.40
Diabetes	0.72 (0.25–1.97)	0.52
Obstructive sleep apnoea	0.82 (0.37–1.81)	0.63
Coronary artery disease	0.45 (0.15–1.26)	0.14
Paroxysmal atrial fibrillation	0.57 (0.27–1.21)	0.14
Long term persistent atrial fibrillation	1.11 (0.21–5.87)	0.90
CHA_2_DS_2_-VASc score	1.32 (0.97–1.83)	0.08
Pulmonary vein isolation plus lines	1.39 (0.68–2.85)	0.36
Cryoablation	1.09 (0.52–2.28)	0.82
Radiofrequency + Cryoablation	0.49 (0.10–2.06)	0.34
Anthracycline exposure	0.62 (0.24–1.54)	0.31
Thoracic radiation	0.37 (0.15–0.86)	0.02
Multiple malignancies	0.89 (0.32–2.31)	0.81

*BMI* body mass index

### Safety outcomes

There was no statistical difference in the incidence of complications within the first 3 months post-ablation, including access and non-access site bleeding, pulmonary vein stenosis, stroke, and cardiac tamponade, between the cancer and non-cancer groups (Table 2).

## Discussion

The results of this study demonstrate that catheter ablation is an effective and safe modality for treating AF in selected patients with cancer. The success rate, defined as freedom from recurrent AF, with or without AAD, and need for repeat ablation at 12 months post-ablation, in patients with cancer was similar to that observed in non-cancer controls. At the same time, safety outcomes, including post-procedural bleeding, pulmonary vein stenosis, stroke, and cardiac tamponade within the first 3 months after catheter ablation, were also similar to non-cancer controls.

There is limited data evaluating the effectiveness and safety of catheter ablation for AF in patients with cancer. A prior propensity-matched cohort study evaluated the effectiveness and safety of cryoablation for AF in 70 patients with cancer and 70 non-cancer controls [20]. In this study, arrhythmia free survival at 12 months did not differ significantly between patients with cancer and controls (67.1 ± 5.8% vs. 77.8 ± 5.1%, *p* = 0.16). Our results agree with and add to the results of this prior study. Importantly, compared to this prior study, our study included a larger number of patients, more patients with active cancer, and both radiofrequency and cryoablation procedures.

The safety of catheter ablation for AF in patients with cancer has been evaluated in 2 prior studies. Eitel et al. evaluated safety outcomes including the development of phrenic nerve palsy, femoral pseudoaneurysms, peri-procedural bleeding, cardiac tamponade, and death and found no difference between cancer and non-cancer patients [20]. In contrast, Giustozzi et al. found a significantly higher risk of clinically relevant bleeding within 1 month after catheter ablation in 21 patients with cancer compared to 163 non-cancer controls [21]. A potential reason for the excess bleeding risk observed by Giustozzi et al. includes their practice of bridging with low molecular weight heparin after the procedure rather than continuing anticoagulation without interruption, as is the usual practice at the institutions included in our study. Furthermore, more than half the patients included in the study by Giustozzi et al. had a history of gastrointestinal and genitourinary malignancies that are more prone to bleeding with anticoagulation than other cancers [22].

In our study, radiofrequency ablation was used more frequently in patients with cancer, while cryoablation was used more commonly in controls. While we do not have data to explain the rationale behind this discrepancy, one possible explanation may be the reduced fluoroscopic exposure with radiofrequency ablation compared to cryoablation. However, both techniques have been shown to be equally efficacious and safe in randomized clinical trials and therefore, it is not surprising that outcomes were similar between cancer and non-cancer controls in our cohort [23]. Additionally, we performed multivariate regression analysis and the type of ablation performed was not identified as a significant predictor of outcomes.

We used multivariable analysis to identify potential predictors of recurrent AF after a 90-day blanking period in patients with and without cancer. BMI was identified as a significant predictor in both groups (Fig. 1). This result is not surprising since obesity has been associated with an increased risk of recurrent AF after ablation in prior studies [24, 25]. Moreover, it has been shown that 10% or more weight loss prior to ablation or bariatric surgery prior to ablation, are both associated with a significant reduction in the risk of recurrent AF [26, 27].

Thoracic radiation therapy for cancer has been postulated to promote inflammation and tissue fibrosis, potentially leading to an increased risk of recurrent AF after ablation. Prior studies have had conflicting results regarding the impact of thoracic radiation therapy for cancer on left atrial scar volume. One study of 7 cancer patients (6 lymphoma and 1 esophageal cancer) treated with thoracic radiation demonstrated a linear relationship between mean cardiac radiation dose and left atrial scar volume on cardiac magnetic resonance imaging [28]. In contrast, another study comparing 38 patients with breast cancer to non-cancer controls did not find any difference in LA scar volumes during electrophysiology mapping [29]. Interestingly, our results did not identify left sided radiation as a significant predictor of recurrent AF in patients with cancer. Similarly, Etial et al. found that arrhythmia-free survival was not reduced in patients with a history of thoracic radiation relative those who did not receive thoracic radiation [20].

Our study has several limitations. Given that this was a retrospective cohort study, we cannot rule out the possibility of selection bias among patients referred for ablation. While this is the largest cohort study to date of cancer patients undergoing catheter ablation for AF, the number of patients is still relatively low, with a small proportion of patients with active cancer (18.3%). Therefore, the true impact of active cancer therapy on the effectiveness, and more importantly, safety of catheter ablation may not be accurately assessed in this study. The types of cancer and cancer therapies were also heterogeneous in our study and further studies are needed to evaluate the effectiveness of catheter ablation for certain cancer therapies, such as Bruton’s tyrosine kinase inhibitors, that are associated with a higher risk of AF. A relatively small number of patients (32%) had received systemic antineoplastic therapy, and only 16% had received anthracyclines. Although given the overall small sample size, a subgroup analysis could not be performed, a multivariable regression analysis of patients with cancer does not identify anthracycline exposure as an independent predictor of AF recurrence. While we have determined the inclusion criteria based on our understanding of the immediate cardiovascular outcomes for patients undergoing cancer treatment and based on the long-term adverse effects of anthracyclines and thoracic radiation therapy, it is important to note that these are somewhat arbitrary and, our knowledge is evolving, especially regarding the novel agents which may be associated with long-term arrhythmogenic effects and our analysis may not account for such effects.

In conclusion, the results of our retrospective cohort study demonstrate that catheter ablation for AF is effective and safe in patients with cancer. The outcomes observed in cancer patients are similar to those seen in patients without cancer, supporting the recommendation that ablation should be offered as a therapeutic modality to treat AF in selected patients with cancer.

## Data Availability

Aggregate de-identified data can be made available on request.
